# Treatment Readiness among Primarily Latine Families Seeking Parent–Child Interaction Therapy (PCIT) in an Urban Setting

**DOI:** 10.3390/ijerph19084784

**Published:** 2022-04-14

**Authors:** Allison Hatley-Cotter, Georgette Saad, Elizabeth Brestan-Knight

**Affiliations:** 1 Department of Psychological Sciences, Auburn University, 226 Thach Ave, Auburn, AL 36849, USA; brestev@auburn.edu; 2 Mary’s Center, Washington, DC 20009, USA; gsaad@maryscenter.org

**Keywords:** parent–child interaction therapy, Latinx, readiness for change, assessment, parenting

## Abstract

(1) Background: Given the high prevalence of childhood mental health problems and their long-lasting negative consequences if left untreated, it is important to investigate factors that affect family engagement in psychological interventions such as Parent–Child Interaction Therapy (PCIT), including caregiver treatment readiness and readiness for change (RFC). Specifically, Latine families experience greater mental health disparities and have unique cultural factors that affect engagement. The current project examined caregiver pretreatment readiness among primarily Latine Spanish- and English-speaking families. (2) Methods: Participants were 100 caregivers (96% female) of young children ages 2 to 7 who sought PCIT services from a community mental health center in Washington, D.C. Families completed written and observational assessment measures at pretreatment and throughout PCIT, which were used for the current study. (3) Results: Caregivers reported high readiness and importance of treatment at intake, with higher RFC among Spanish-speaking caregivers. Regardless of language, caregivers who reported more frequent and problematic child misbehavior and who were from a multi-caregiver household tended to report more RFC and treatment importance at intake. Pretreatment RFC also predicted family completion of the first phase of PCIT although there was a high attrition rate for the sample as only 18% of families completed treatment. (4) Conclusions: These findings provide insight into the implementation of standard PCIT among Spanish- and English speaking families and highlight the benefits of assessing pretreatment caregiver readiness to inform clinical decision-making.

## 1. Introduction

Estimates for the prevalence of childhood mental health problems range from 13 to 40 percent, with associated negative outcomes related to academic, social, health, and occupational functioning [1,2,3,4,5]. Despite growing evidence for empirical support and increased dissemination efforts, only approximately half of children with mental health problems obtain treatment even among those with severe impairment [6,7,8]. Moreover, up to 60 percent of families who do seek psychological intervention drop out prematurely and receive an inadequate dosage of treatment [9,10]. Premature termination of services may result in reduced treatment benefits and losses in mental health resources, such as personnel and financial losses [11,12,13]. As such, understanding factors that affect family readiness and willingness to engage in mental health treatment is vital, particularly for young children who rely heavily on caregivers for treatment engagement [13,14].

Certain family characteristics have been found to be associated with initial and continued engagement in treatment services, including low socioeconomic status (SES), stress, single parenthood, and ethnic minority status [15,16,17]. Families who identify as Black, Indigenous, and People of Color (BIPOC) are more likely to drop out of treatment prematurely, less likely to engage in mental health services, and more likely to experience mental health disparities [16,18,19]. In particular, numerous mental health disparities exist for Latine families, including higher unmet needs for mental health care, higher odds of being uninsured, and lower odds of mental health visits even though environmental factors place Latine youth at a greater risk for developing mental health problems [18,20]. Latine families are more likely to receive lower quality of care through reduced access to evidence-based interventions and negative interactions with health-care providers [21,22,23]. Moreover, cultural and community-based factors, such as discrimination and mental health stigma, influence Latine families’ willingness to engage in treatment and subsequent therapy outcomes [21,22,24]. Language appears to impact treatment utilization as Spanish-speaking Latine caregivers have lower levels of engagement in public health services and poorer perceptions of health services compared to English-speaking Latine and non-Hispanic white families [25,26].

To address these mental health disparities, some intervention programs have culturally adapted their protocols to better align with the values and context of Latine families [27,28,29,30]. Parent–Child Interaction Therapy (PCIT), an empirically supported parent training program, has pilot-tested two adapted protocols for Latine families, incorporating culturally relevant factors like extended session time, inclusion of extended family members, and increased orientation to treatment [31,32,33,34]. Training “natural helpers” to disseminate PCIT and utilizing incentives within treatment have also been explored to better reach and engage Latine families [35,36]. Thus far, results have shown greater improvements in child and parent behaviors compared to treatment as usual among Mexican American and Puerto Rican families; however, culturally adapted PCIT protocols have not consistently shown better outcomes or reduced attrition than standard PCIT [29,32,37,38]. Further investigation of factors that influence engagement and outcomes for Latine families in PCIT is needed, particularly given variability in cultural values, language, and level of acculturation within this population [33,39,40]. In fact, one recent study found that Spanish- and English-speaking Latine families have similar acquisition of skills during PCIT but different utilization based on language [41]. Language has also been shown to interact with other factors, such as advertising messenger, to predict caregiver attitudes towards help-seeking with PCIT [42]. Such findings highlight the complexity and importance of understanding how cultural, attitudinal, and other characteristics of Latine families influence their experience of and engagement in PCIT.

Caregiver readiness for change (RFC) and for treatment is one factor believed to affect engagement that has not yet been examined among Latine families within the context of PCIT [43,44,45]. Caregivers presenting for therapy services often vary in their understanding of the treatment process, initial skill level, and motivation for change, influencing their readiness to engage in treatment and to change their behavior [46]. Research has shown that caregivers who report higher levels of RFC are less likely to drop out of treatment and tend to remain in treatment longer compared to those with lower pretreatment RFC [47]. Exploring caregiver readiness at intake could help clinicians to promote engagement and prevent attrition with motivation enhancement strategies, particularly for BIPOC families [15,16,19,48]. For Latine families, caregiver readiness may be influenced by various factors, such as but not limited to mental health stigma, level of acculturation, misconceptions about treatment, and caregiver’s experiences with their clinician [24,49,50].

### Goals and Hypotheses of the Current Study

The current study aimed to (1) describe the experience of Spanish- and English-speaking Latine families within PCIT treatment, (2) examine caregiver RFC within this population, and (3) investigate factors impacting RFC and the influence of caregiver readiness on PCIT retention. To add to the literature on Latine families participating in PCIT, descriptive information related to child and family characteristics, RFC, and treatment engagement was examined and compared to previous studies. Based on previous language-based differences in engagement and perceptions of health services, we hypothesized that Spanish-speaking caregivers would report lower levels of parental readiness at pretreatment compared to English-speaking caregivers [25,26]. Second, associations between pretreatment caregiver readiness and child and family characteristics were investigated, including caregiver-reported child behavior problems, observed caregiver behaviors, and demographic information (i.e., caregiver age, length of residency in the US, household status). It was hypothesized that caregivers with higher ratings of child behavior problems, lower observed use of negative or leading verbal behaviors (e.g., commands, negative talk), and higher rates of positive verbal behaviors (e.g., praise, reflection) at their intake would endorse greater readiness for treatment based on previous research [51,52,53,54]. We also predicted that caregivers from single-parent households, who were younger, and who had lived in the US for less time would report lower levels of readiness [15,25,26]. Finally, the association between caregiver RFC at pretreatment and PCIT retention was investigated. We hypothesized that caregivers who endorsed greater readiness at intake would attend more PCIT sessions compared to those with lower readiness scores [53].

## 2. Materials and Methods

### 2.1. Participants

Participants were 100 primary caregivers of children who received PCIT services through the Behavioral Health Department at Mary’s Center, a Federally Qualified Health Center (FQHC), in Washington, D.C. For the project, families who met criteria for a PCIT referral, initiated therapy services, and provided informed consent were included in the study. Caregivers were majority female (96%) and Latine (85%) with a mean age of 32.41 years (SD = 8.53). About half (54%) of the caregivers who participated spoke Spanish as their primary language. Children in PCIT were majority male (67%) with a mean age of 4.70 years (SD = 1.37). For families who disclosed income data (about 50% of the sample), most reported an annual income of less than USD 40,000 (94.2%). Comparing demographics based on language, English-speaking caregivers were significantly more likely to be in a single-parent household and less likely to have a female child in treatment compared to Spanish-speaking caregivers, χ^2^ (1) = 3.96, *p* = 0.047 and χ^2^ (1) = 4.89, *p* = 0.027. English-speaking caregivers also reported significantly longer residency in the US compared to Spanish-speaking caregivers, *t* (34) = 6.68, *p* < 0.001. Demographic characteristics are reported in Table 1.

### 2.2. Procedures

Families initiating PCIT services at Mary’s Center had the opportunity to provide informed consent to participate in the IRB-approved research study during intake procedures. The project was approved by the Auburn University IRB (protocol code 17-001 EP 1702). Approximately half (49.35%) of the families provided informed consent for researchers to use their treatment information during the data collection period. Regardless of their research involvement, all families attended an intake that involved completion of administrative forms and diagnostic assessment measures, participation in a Dyadic Parent–Child Interaction Coding System (DPICS) observation, and engagement in treatment planning with a clinician [55]. After completing the intake, families participated in the standard PCIT protocol, which was conducted in the caregiver’s preferred language. PCIT has two treatment phases: Child-Directed Interaction (CDI) aims to improve parent–child relations, and Parent-Directed Interaction (PDI) aims to increase child compliance and caregiver consistency with discipline [31]. Caregiver-reported and observational assessment measures are conducted at each PCIT session to track family progress. Caregivers must meet goal skill criteria to move from CDI to PDI (i.e., using 10 labeled praises, 10 behavior descriptions, and 10 reflections in a 5 min period) and to complete PCIT (i.e., 75% effective commands with correct follow through and ECBI score less than 114) [31]. Assessment and treatment data for all families were entered into an Electronic Medical Record (EMR) throughout PCIT. For the current study, de-identified data were pulled from the EMR and entered into a database for caregivers who provided informed consent to participate in research.

### 2.3. Measures

#### 2.3.1. Demographic Information

Demographic information was collected for each family during the intake process. For the current study, the following information was used to describe the sample and for analyses: the age and gender of the target caregiver and child as well as the caregiver’s ethnicity, single or multi-parent household status, income, length of residency in the US, and preferred language.

#### 2.3.2. Readiness, Efficacy, Attributions, Defensiveness, Importance Scale, Short Form (READI-SF)

The READI-SF is a 17-item self-report measure of caregiver RFC, treatment readiness, and perceived importance of treatment [53]. Caregivers rate their agreement with statements, such as “I want to change the way I discipline my child,” and “I am willing to do whatever it takes to be sure that we get help,” on a 5-point scale (1—Strongly Disagree, 5—Strongly Agree). Responses for each item can be summed to create a total readiness score and several subscales, with higher scores indicating greater readiness [53,56]. Research has shown adequate to good internal consistency for the READI-SF among community and clinic-referred samples as well as initial support for convergent, discriminative, and predictive validity [53,56,57,58]. In the current study, a 13-item version of the READI-SF was utilized based on factor analysis, with two subscales: Readiness for Change (RFC) and Treatment Importance [59]. The READI-SF was translated into Spanish by Mary’s Center staff for the study using the back translation method [60,61]. Translators intended for the measure’s language to be universal across Spanish-speaking ethnic groups and simple for caregivers with lower reading abilities while still maintaining the READI-SF’s integrity. During their intake, caregivers elected to complete either the English or Spanish version of the READI-SF. Both the RFC Scale and the Treatment Importance Scale demonstrated good internal consistency for our sample (α = 0.87, α = 0.73).

#### 2.3.3. Eyberg Child Behavior Inventory (ECBI)

During the intake, caregivers completed either the Spanish or English version of the ECBI, a 36-item parent-report measure of current child behavior problems, which has been validated for children ages 2 to 16 [62]. Items assess common behavior problems, such as “argues with parents about rules,” “has short attention span,” and “cries easily.” The ECBI has two scales: the Intensity Scale and the Problem Scale. For the Intensity Scale, parents rate how frequently each behavior occurs on a scale from 1 (Never) to 7 (Always). For the Problem Scale, parents respond to the question “Is this a problem for you” by circling YES or NO for each item. Higher scores indicate more frequently occurring and problematic child behaviors, with clinical cutoffs based on normative data [62]. The ECBI scales have demonstrated good internal consistency, inter-parent agreement, test–retest reliability, convergent validity, and discriminative validity [62]. It has been translated into Spanish with evidence for internal consistency, test–retest reliability, and concurrent validity [63]. For the current sample, Cronbach’s alpha was 0.95 for the ECBI Intensity Scale; this could not be calculated for the Problem Scale because only total scores, not item responses, were available from the EMR data for this scale.

#### 2.3.4. Dyadic Parent–Child Interaction Coding System, Fourth Edition (DPICS-IV)

The DPICS is a behavioral observation measure used to assess the quality of parent–child interactions in PCIT [55]. During the DPICS, parent–child dyads are typically alone in a clinic playroom, with a clinician providing instructions to the caregiver using a remote communication device. The frequency of parent and child verbalizations are coded by the clinician during three different structured play situations: child-led play, parent-led play, and clean up. This situational arrangement leads the caregiver to exert more control over the child’s behavior throughout the observation [55]. The DPICS-IV has nine parent coding categories. DPICS coding categories have demonstrated high to adequate inter-observer agreement, convergent validity with related constructs, discriminant validity for clinic-referred and non-clinic referred families, and treatment sensitivity for changes in parent–child interactions [55]. For the current study, caregiver behaviors coded across all three segments in the pretreatment DPICS observation were combined, and three composite coding categories were calculated and used for analyses: positive caregiver verbalizations (behavior description, labeled praise, unlabeled praise, and reflection), caregiver demandingness (direct commands/total commands), and inappropriate caregiver verbalizations (question, negative talk, direct command, and indirect command) within the context of PCIT.

#### 2.3.5. Treatment Retention

Treatment retention was evaluated based on the number of PCIT-related sessions that each family attended. Given that PCIT is not time limited and treatment length can depend on several factors (e.g., difficulty learning skills, more challenging child behavior), family completion of CDI was also examined.

### 2.4. Analyses

Analyses were conducted using SPSS. To manage missing data, multiple imputation was conducted, and imputed datasets were used for analyses, providing pooled results for compatible tests [64,65]. For our first hypothesis, *t*-tests were run to examine mean differences in responding on the READI-SF scales between English-speaking and Spanish-speaking caregivers. For our next hypotheses, linear regression analyses were run to examine associations between pretreatment caregiver readiness and child and family characteristics. The RFC and Treatment Importance Scales were entered as dependent variables; the two ECBI scales, three DPICS composite codes, and relevant demographic characteristics were entered as independent variables; and caregiver language was entered as a covariate for these analyses. For our final hypothesis, multiple linear and binary logistic regression analyses were run to determine whether caregiver-rated RFC or treatment importance predicted treatment retention based on number of sessions attended and completion of CDI after accounting for language.

## 3. Results

On average, caregivers reported that their children exhibited clinically significant and problematic behavior on the ECBI Intensity (M = 142.57, SE = 4.56) and Problem Scales (M = 18.33, SE = 0.94) during the intake. Regarding observed caregiver behavior at pretreatment, caregivers exhibited a high frequency of directiveness (M = 58.95, SE = 3.03) and inappropriate behavior (M = 64.77, SE = 4.00) with comparably low levels of prosocial behavior based on DPICS coding composite categories (M = 10.36, SE = 1.08). No significant differences in pretreatment ECBI ratings or DPICS codes were found based on caregiver language. At intake, caregivers generally reported high RFC (M = 26.04, SE = 0.44) and treatment importance (M = 30.12, SE = 0.40) on the READI-SF. Pooled results for *t*-tests indicated that Spanish-speaking caregivers reported significantly higher scores on the RFC scale (M = 27.21, SE = 0.41) compared to English-speaking caregivers (M = 24.66, SE = 0.78), *t* = −2.88, *p* = 0.004. There was no significant difference between reports of Spanish-speaking (M = 30.79, SE = 0.45) and English-speaking caregivers (M = 29.32, SE = 0.67) on the Treatment Importance scale, *t* = −1.82, *p* = 0.069.

When examining factors associated with caregiver readiness, household membership and caregiver reports of problematic child behavior as measured by the ECBI Problem Scale were the only significant predictors of caregiver RFC, *t* = −2.21, *p* = 0.027, and *t* = 2.83, *p* < 0.01. At intake, caregivers living in a single parent household reported lower RFC compared to those in a multi-caregiver home, whereas caregivers who rated their child’s behavior as more problematic reported higher RFC compared to those with lower ECBI problem scores. Both the ECBI Intensity and Problem Scales were significant predictors of caregivers’ perceived importance of treatment after accounting for language, *t* = 4.67, *p* < 0.01 and *t* = 4.30, *p* < 0.01. None of the DPICS composite categories or other caregiver characteristics (e.g., age, years of residency in the US) were significant predictors for either caregiver RFC or treatment importance (Table 2 and Table 3).

In our sample, 12 families were still participating in treatment when data were analyzed, so they were excluded from remaining analyses. On average, caregivers reported completing the assigned home practice about half of the days each week throughout PCIT (M = 55.21%, SD = 26.38%). Thirty-three families (37.5%) demonstrated CDI skill acquisition to progress to PDI, and 16 families (18.2%) graduated from PCIT. The mean number of sessions attended was 16.69 (SD = 9.67) for families who completed PCIT and 7.56 (SD = 6.69) for families who terminated treatment early. The percentage of caregivers who met CDI goal criteria and PCIT graduation criteria did not differ based on their preferred language, χ^2^ (1) = 0.028, *p* = 0.87 and χ^2^ (1) = 0.26, *p* = 0.61. When examining associations between caregiver readiness and treatment retention, neither the RFC scale nor the Treatment Importance scale significantly predicted the number of PCIT sessions attended (*t* = 1.00, *p* = 0.32; *t* = −0.45, *p* = 0.65). Caregiver pretreatment reports on the RFC scale, but not the Treatment Importance scale, were marginally significant in predicting whether families completed CDI or not regardless of caregiver language (*p* = 0.085).

## 4. Discussion

A large percentage of youth with mental health problems do not obtain psychological treatment or drop out of treatment prematurely due, in part, to lack of caregiver engagement in services [7,8,9,10]. Specifically, Latine families face both practical and cultural barriers to accessing mental health services, impacting their initiation, engagement, and retention in treatment [15,24,25,33,49]. The dissemination and cultural adaptation of PCIT has demonstrated preliminary support for positive outcomes among Latine families; however, more nuanced understanding of factors impacting PCIT experience, engagement, and retention for Latine families is need [37,38]. As such, the current project aimed to describe the treatment experience and to examine caregiver readiness and perceived importance of treatment among primarily Latine families seeking PCIT in a community mental health clinic.

Within our sample, caregivers reported mean ECBI Intensity and Problem scores that were above the clinical cutoff at intake, consistent with community-based PCIT studies of English-speaking families [66,67]. No differences in ECBI ratings were found based on caregiver language, suggesting that the Spanish- and English-version of the ECBI can detect clinically relevant child behavior problems as long as caregivers are able to complete the measure in their preferred language. During the pretreatment DPICS observation, caregivers were observed to utilize fewer prosocial behaviors as well as more commands and inappropriate behaviors, as classified through the PCIT framework, when interacting with their child. However, these results are consistent with observations of Latine and non-Latine caregivers in other PCIT studies, suggesting that caregivers generally do not utilize PCIT skills measured by the DPICS (e.g., labeled praise, behavior descriptions) at pretreatment [68,69]. Interestingly, there were no mean differences in observed behaviors between Spanish- and English-speaking caregivers during the DPICS, which is inconsistent with previous findings that Spanish-speaking caregivers utilize more commands and questions at intake and throughout treatment [41]. These findings have implications for the evaluation and interpretation of caregiver behavior within PCIT for Spanish-speaking families, particularly since treatment progress is partially based on changes in caregiver behavior [31].

Only 16 families (18%) completed PCIT in our sample, for an attrition rate of 82%. This level of dropout is higher than previous PCIT studies of Latine families (10–43%) but is closer to rates found for PCIT implemented in community mental health settings (67–80%) [29,32,37,67,69,70,71]. Consistent with previous research, a larger percentage of families dropped out early in treatment (38.2% before CDI coaching sessions) and during CDI (24.7%) compared to those that dropped out during PDI (19.1%) [36,67,69]. Families in our sample appeared to be in a lower SES, which may account for the higher rate of treatment dropout observed [51,54,72]. Data regarding discharge reasons were not available for our sample, so it may be that families who did not graduate from PCIT had to terminate for unavoidable reasons (e.g., moving out of state) or it may be that they received sufficient clinical benefits without reaching graduation criteria [31,51]. In fact, research suggests that children exhibit behavioral improvements within the first four sessions of PCIT, which may partially explain the higher dropout rate observed in CDI [73]. This attrition rate may also conflate families who terminated PCIT services prematurely with those who were referred for other services within Mary’s Center during treatment (e.g., individual therapy for parent, different treatment modality) given the availability of several mental health options within this FQHC. In fact, the “need for different/more intensive services” was one of the top reasons endorsed for dropout in another community-based PCIT study, suggesting that families who prematurely terminate PCIT services may still engage in mental health treatment to address current concerns [74]. Interestingly, caregivers’ preferred language was not significantly related to their progression through PCIT (i.e., acquisition of CDI/PDI goal skill criteria). Research suggests that Latine families tend to have less access to quality mental health services; however, it appears that when evidence-based treatments are available and provided using their preferred language, Spanish- and English-speaking Latine families are equally likely to remain in PCIT based on our results [21,22,23]. Notably, all clinicians providing services in this study were bilingual, and many bicultural, which may enhance clinicians’ ability to engage caregivers as they were able to utilize their preferred language throughout PCIT.

In general, families within our sample reported high RFC and treatment importance when initiating PCIT. As expected, average caregiver readiness in this sample was higher compared to a community-based sample [53]. Families initiating mental health services have likely overcome barriers and invested time and resources to attend an intake session, potentially accounting for higher RFC and treatment importance compared to families who have not yet engaged in treatment [14,48]. These findings are particularly promising because Latine families have been shown to report negative attitudes and expectations about whether therapy services will be effective or helpful [75]. Contrary to our hypotheses, Spanish-speaking caregivers had higher scores on the RFC scale when starting PCIT compared to English-speaking caregivers. Research has previously shown a relationship between language status, poorer perceptions of health services, and lower levels of engagement in services among Latine families. However, it is also possible that cultural values, such as respeto (respect for authority) and simpatia (emphasizing warm and positive interactions that avoid conflict), affected Spanish-speaking caregiver ratings on the READI-SF, as they may be less acculturated than English-speaking caregivers [25,26,76]. In fact, some studies have shown that Latine families who are less acculturated endorse fewer barriers and have lower rates of “no shows’’ to mental health appointments, suggesting complexity in how culture relates to mental health services utilization and engagement [75,77]. Given that the Latine population is not a homogeneous group, it is worthwhile to note that mental health use has been shown to differ depending on Latine subgroup [49]. Limited availability of mental health resources for Spanish-speaking families may also relate to caregiver treatment readiness, contributing to greater readiness when families are able to receive services in their preferred language [18,25,26]. Overall, these findings suggest that caregivers who do endorse lower readiness at intake may benefit from tailored, culturally attuned engagement strategies. Anecdotally, some caregivers commented during intake calls that they wished an appointment had been scheduled when they were first referred to treatment and that their child’s behavior had improved slightly since then. The length of time between referral and treatment initiation as well as changes in child symptoms while families are on a waitlist may relate to a caregiver’s urgency and readiness when they are able to begin treatment.

Some child and family characteristics were associated with caregiver readiness at intake even after accounting for language. Consistent with our hypotheses, caregivers who reported more frequent and problematic child behavior problems tended to report greater readiness to change behavior and importance of receiving treatment at pretreatment [52,53,54]. Notably, the ECBI Problem scale was significantly associated with both READI-SF scales, whereas the Intensity scale was only associated with the Treatment Importance scale. This suggests that caregivers who report more frequent child behavior problems may feel that they are not able to manage their child’s behavior independently and, thus, recognize the importance of seeking help. However, they may not fully understand the relationship between their child’s behavior problems and their own parenting behavior, affecting caregiver-reported readiness to change their own behavior. For example, these parents may attribute their child’s conduct problems to internal attributes rather than their own parenting behaviors, which is more likely for families of children referred for conduct problems [78,79]. Furthermore, while the ECBI Intensity Scale measures the frequency of child behavior problems, the ECBI Problem Scale is often considered an indirect measure of caregivers’ tolerance of misbehavior or stress [80,81]. Prior research has shown that caregiver stress moderates the relationship between caregiver readiness and child behavior problems [57]. As such, caregivers with higher ratings on the ECBI Problem Scale may be experiencing high levels of stress, which makes them more willing to endorse readiness for behavior change as well as importance of seeking treatment [57]. On the other hand, Spanish-speaking and English-speaking caregivers who do not report that their child’s behavior is problematic or who report less frequent conduct problems are likely to feel that treatment is less important and to be less ready to change their behavior, which could impact treatment outcomes.

Household status (e.g., single caregiver household or not) was the only other significant predictor of caregiver readiness to change their behavior although it did not predict perceived importance of treatment. Consistent with our hypothesis, single caregivers tended to report lower levels of RFC compared to those who had another caregiver living in the household [15,25,26]. Given that single parent status has been linked to treatment dropout, our results suggest that RFC may partially account for this relationship [15,82]. Thus, single caregivers entering PCIT may be less ready to put effort into changing their parenting and may benefit from greater attention to treatment barriers, motivation enhancement, and support from their therapist early in treatment when families are at a higher risk for dropping out [67,69]. Still, findings should be interpreted with caution because data on household status were only available for part of our sample, and some previous studies have shown different directions for this relationship [43]. Otherwise, no significant associations were found between caregiver readiness, observed caregiver behaviors, or caregiver/family characteristics, contrary to our hypotheses. Although research suggests that caregiver behavior at pretreatment predicts treatment dropout, it does not appear that caregiver readiness contributes to this association [51]. Rather, it may be that caregiver perceptions regarding child behavior and other areas (e.g., parenting competence, self-efficacy) at pretreatment relate to their readiness, which has been supported in some previous research [82].

Though only marginally significant, caregivers who reported that they were more ready to put effort into changing their behaviors at intake were also more likely to complete the CDI phase of PCIT even after accounting for caregiver language. For PCIT, parents may experience decreased motivation during CDI if they believe that it is not relevant to their problems (e.g., it does not address discipline directly) or if they have difficulty mastering the skills. Moreover, families generally experience reductions in behavior problems during CDI, which could either increase treatment readiness (e.g., they see the effects and are motivated to continue improving) or decrease treatment readiness through the “CDI cure” (e.g., they believe their problems are solved so continued treatment is not necessary) [51,67,83]. Given that most families dropped out of treatment during CDI, assessing caregiver readiness for change at the start of PCIT appears to predict retention through this first phase of treatment, providing clinicians with insight into which families may benefit from additional motivation enhancement, assessment of barriers, and tailoring to fit their needs. This insight may be particularly helpful as research suggests that the influence of motivational interventions used prior to treatment depends on caregivers’ pretreatment level of readiness as caregivers with low pretreatment readiness benefit the most [84]. In previous research, there have been mixed results regarding the association between initial child behavior problems and treatment dropout, with some studies showing that higher caregiver-reported child behavior problems predicting treatment dropout [85]. Our findings indicate that caregivers who endorse more child behavior problems are likely to have greater pretreatment readiness, which subsequently predicts treatment retention through CDI, suggesting that this may serve as a source of motivation rather than a barrier for remaining in PCIT [82]. Although number of sessions attended has been shown to relate to PCIT graduation, caregiver readiness did not predict the number of PCIT sessions that families attended even though it predicted CDI completion [73,74]. Our results suggest that using the number of sessions attended as a measure of treatment retention for PCIT may not exclusively capture the intended construct as families may be attending more sessions due to difficulty acquiring skills, more severe child conduct problems, or lack of confidence in applying therapy techniques, which all factor into progressing through and completing PCIT [13,31].

### 4.1. Limitations

The current study has some limitations. First, families in our sample were referred for treatment and self-selected into the research project. As such, they may have unique qualities that are not generalizable to all individuals [86]. Second, similar to other studies of readiness, caregivers had high ratings on the READI-SF indicating a ceiling effect, which may have restricted the range of responses and affected power to detect significant relationships for some variables [14,48,87]. We also used a shortened version of the READI-SF with two subscales, which differs from previous research [53,58]. Third, our study utilized language as a proxy for culture but did not specifically measure caregiver level of acculturation or beliefs. As such, we were not able to test theory-based explanations relating to culture or level of acculturation. Additionally, we were not able to separate the influence of SES from ethnic minority status because most families in our sample were low income and Latine, limiting our understanding of specific effects for these variables. Finally, no treatment fidelity or DPICS coding reliability information were available in this study. Although research suggests that clinicians within community mental health settings are able to implement PCIT with relatively high fidelity, we were not able to examine this aspect of implementation [88].

### 4.2. Future Directions

Despite these limitations, this study offers support for the implementation of the standard PCIT protocol conducted in Spanish, which is a relatively underexplored circumstance [41]. In fact, Latine families tend to be the most underrepresented group in studies examining psychological interventions, limiting support and understanding of treatment effects within this population [89,90]. Future research should continue examining the effectiveness of standard and culturally adapted PCIT protocols within BIPOC families to determine if there are unique benefits for specific cultural adaptations and to inform clinician decision-making for treatment tailoring. At post-treatment, Latine caregivers have previously been shown to endorse high treatment satisfaction and positive attitudes towards PCIT, with similar ratings compared to non-Latine families; however, identification of specific factors that contribute to positive PCIT experiences for Latine families is still needed, such as caregiver readiness, language, components of cultural adaptations, and client–therapist matching [29,37,91].

Moving forward, additional attention should also be paid to tailoring engagement in treatment to support access to standard PCIT for families that identify as Latine who speak English or Spanish. Providing empirical support for language-based aspects of PCIT, such as goal skill criteria and therapist coaching techniques, will be important to ensure that these aspects do not differentially impact Spanish-speaking families [41]. Additionally, investigating the association between readiness and factors, such as level of acculturation, cultural beliefs, ethnic group identity, and perceptions of mental health treatment, may enhance clinical decision-making regarding tailoring treatment to fit family needs [33]. In this study, clinicians represented an exclusive group who were able to provide PCIT services in English and Spanish, as only 5.5% of current clinicians are able to provide services in Spanish based on a nationwide survey by the American Psychological Association [92]. In many cases, the clinicians in this study also identified as bicultural. Future research should explore the interaction between therapist and family characteristics, such as coaching style, cultural beliefs, and language, particularly given the amount of diversity within Latine populations [41,49]. Future directions may involve assessing cultural beliefs about treatment, beliefs about the origin of the presenting problem, and how a bicultural identity of a provider may impact engagement in the Latine community.

In addition, future studies should explore whether assessing caregiver readiness at different time points in the treatment process would provide more informative responding. For example, incorporating the READI-SF into telephone screening for potential clients might detect greater variability in caregivers’ readiness to begin treatment because clients have not yet invested time and resources to meet in person with a clinician. Given that families can wait weeks or longer on clinic waitlists, it may also be helpful to assess how caregiver readiness changes from when a family is referred, when an appointment is offered, and when families attend an appointment. Moreover, it may be important to assess readiness at intake and after several treatment sessions to better understand how caregivers respond to therapy techniques or to introduce additional opportunities after sessions to create space for a feedback loop from caregivers [14]. Thus, future research should focus on understanding how caregiver readiness changes across treatment, how this relates to treatment retention, and how cultural factors impact these associations among BIPOC families. This information could help clinicians know when to utilize measures of caregiver readiness and how to interpret caregiver responses to inform treatment planning.

## 5. Conclusions

Our study provides insight into the experience and caregiver readiness of Spanish- and English-speaking Latine families seeking PCIT within a community-based mental health setting, a population with greater mental health disparities and risk factors for treatment dropout [15,18,20]. Uniquely, families in this sample were able to receive assessment and treatment services in their preferred language, eliminating one influential treatment barrier. Overall, caregivers endorsed high levels of treatment importance and RFC, with higher ratings among Spanish-speaking caregivers, contrary to our hypothesis. Consistent with our hypotheses, some factors were related to caregiver readiness and treatment importance, including child behavior problems and household status. Still, not all hypothesized relationships were observed (e.g., observed caregiver behavior, caregiver age). Pretreatment RFC also appeared to predict retention through the first phase of PCIT, providing partial support for our original hypothesis. Based on these findings, assessing caregiver readiness may help clinicians decide whether additional rapport-building activities or motivation-enhancement techniques are needed for Latine families to remain engaged in treatment. Still, our project highlighted several difficulties with conducting research and implementing evidence-based treatments in community-based mental health settings, particularly given the high attrition rate within our sample. These results provide valuable areas upon which future research can build.

## Figures and Tables

**Table 1:** Demographic characteristics for parent–child dyads.

Demographic Variable		M/SD	N	%
Caregiver Age		32.41/8.53	75	
Missing			25	
Caregiver Gender	Female		96	96.0
	Male		4	4.0
Child Age		4.70/1.37	100	
Child Gender *	Male		67	67.0
	Female		33	33.0
Ethnicity	Caucasian		5	6.3
	African American		5	6.3
	Latine		68	85.0
	Asian		2	2.5
	Missing		20	
Family Annual Income	<USD 20,000		15	28.8
	USD 20,000–40,000		34	65.4
	>USD 40,000		3	5.8
	Missing		48	
Single Parent Household *	Yes		26	47.3
	No		29	52.7
	Missing		45	
Caregiver Length of Residence in US *		17.14/13.02	36	
Missing			64	
Caregiver Primary Language	English		46	46.0
	Spanish		54	54.0

Note: * = significant difference based on preferred language; M = mean; SD = Standard Deviation; N = Number of families; % = Percentage of families; Percentages were calculated based on the information available (excluding missing cases).

**Table 2:** Results from Linear Regression Analyses for ECBI Scales and DPICS Codes with Language as Covariate.

Dependent Variable	Independent Variables	Unstandardized B	Standard Error	*t*	*p*
RFC	ECBI Intensity	0.013	0.012	1.06	0.29
	Language	2.71	0.86	3.16	**0.002**
RFC	ECBI Problem	0.16	0.057	2.83	**0.005**
	Language	2.52	0.81	3.12	**0.002**
RFC	DPICS Prosocial	0.005	0.053	0.095	0.93
	Language	2.55	0.86	2.97	**0.003**
RFC	DPICS Directiveness	−2.23	1.93	−1.16	0.25
	Language	2.71	0.86	3.15	**0.002**
RFC	DPICS Inappropriate	−0.008	0.014	−0.57	0.57
	Language	2.51	0.85	2.94	**0.003**
Importance	ECBI Intensity	0.043	0.009	4.67	**0.0001**
	Language	2.033	0.72	2.84	**0.005**
Importance	ECBI Problem	0.19	0.045	4.30	**0.0001**
	Language	1.44	0.72	1.99	**0.046**
Importance	DPICS Prosocial	0.003	0.049	0.061	0.95
	Language	1.48	0.80	1.85	0.064
Importance	DPICS Directiveness	−1.70	1.84	−0.93	0.35
	Language	1.60	0.80	2.00	**0.046**
Importance	DPICS Inappropriate	−0.016	0.013	−1.20	0.23
	Language	1.38	0.79	1.74	0.081

Note: **Bold** = *p* < 0.05; RFC = Readiness for Change scale; ECBI = Eyberg Child Behavior Inventory; DPICS = Dyadic Parent–Child Interaction Coding System.

**Table 3:** Results from Linear Regression Analyses for Demographic Variables with Language as Covariate.

Dependent Variable	Independent Variables	Unstandardized B	Standard Error	*t*	*p*
RFC	Parent Age	0.025	0.045	0.57	0.57
	Language	1.34	0.77	1.74	0.082
RFC	Parent Years in US	0.052	0.062	0.84	0.40
	Language	3.08	1.63	1.89	0.059
RFC	Single Parent Household	−2.48	1.13	−2.21	**0.027**
	Language	2.49	1.13	2.20	**0.028**
Importance	Parent Age	0.034	0.054	0.64	0.53
	Language	1.82	0.94	1.95	0.052
Importance	Parent Years in US	0.001	0.080	0.017	0.99
	Language	2.14	2.10	1.02	0.31
Importance	Single Parent Household	0.70	1.11	0.63	0.53
	Language	2.59	1.11	2.33	**0.020**

Note: **Bold** = *p* < 0.05; RFC = Readiness for Change scale.

## Data Availability

Data are available upon request to the corresponding author.
